# Is human DNA enough?—potential for bacterial DNA

**DOI:** 10.3389/fgene.2013.00282

**Published:** 2013-12-13

**Authors:** Sarah L. Leake

**Affiliations:** School of Criminal Justice, Institut de Police Scientifique, University of LausanneLausanne, Switzerland

**Keywords:** forensic, saliva, microbiome, interpretation, human Identification

Human identification has played an important role in forensic science for the past two decades and it will continue to do so. However, there are certain types of traces, for example, low quality and low quantity of DNA, often associated with violent crimes, which cannot always be satisfactorily exploited by current techniques. So what is next? Do we try to push these techniques beyond their limit or do we look to something else? I propose turning to a new source of information—bacterial DNA. I do not suggest bacterial DNA analysis will replace standard DNA typing but it would be a complimentary technique for when the latter provides only limited information (Leake, 2012).

Since the 1980's, there has been a considerable increase in the capacity of human DNA analyses to contribute to the process of individualization. With advances in technology two new breakthroughs, in the late 80's to early 90's, changed the techniques used for DNA analysis. The first, a new marker for DNA analysis, the microsatellite or Short Tandem Repeat (Jeffreys et al., 1985). The second, a new method of visualization based on fluorescent labeling which when combined with PCR increased the sensitivity of the technique enabling low quantities of DNA to be analyzed (Frégeau and Fourney, 1993). Improved sensitivity extends the methods to traces with low template level material and traces containing degraded DNA. A number of techniques exist to help exploit such traces; Y-STRs, mini-STRs, and mitochondrial DNA (mtDNA). The first two exploit nuclear DNA so are subject to the same constraints as standard DNA typing. mtDNA is found in much higher quantities than nuclear DNA and is thus, well adapted for analyzing degraded DNA. However, it is different to nuclear DNA in that results are less informative for a particular person; instead, they typically characterize a maternal lineage. Therefore, it is of continuing interest to think about novel ways to exploit forensic samples to compliment current methods. I propose the analysis of parts of the human microbiome, in particular saliva. This will be accompanied by challenges in interpretation, such as the combination of evidence (i.e., standard DNA typing with results of microbiome analyses), which thus, represents a field that should receive further attention (Juchli et al., 2012).

What is the so-called human microbiome? In brief, the human microbiome describes all the microbiomes found within and across the human body (Turnbaugh et al., 2007). Each distinct area of the human body (for example, the oral cavity, forearm, hand, and gut) have their own microbiome. Each microbiome consists of different combinations of bacteria with, in theory, each person having a slightly different ratio or combination of bacteria at each site. Fierer et al. (2010) investigated the use of bacteria for human identification concentrating on the potential of analyzing skin bacterial communities. They suggested that the bacteria left behind after touching a surface could be used to trace it back to its source. The analysis of the whole salivary microbiome has not yet been applied to forensic science. However, Kennedy et al. (2012) investigated the microbial analysis of bite marks, specifically streptococcal DNA, in order to compare bacteria in the bite mark to those of a potential source. They concluded that this was a feasible comparative analysis and the results could also provide valuable information when the perpetrator's DNA cannot be recovered. Saliva is commonly found at crime scenes and is often transferred from the perpetrator to the victim, especially in sexual assault cases. Due to a number of factors including environmental, poor DNA transfer and the major contributor masking the minor contributor, human DNA analysis does not always work, demonstrating the need for an alternative technique. One of the major advantages of bacteria is that they are more resistant to environmental factors than human DNA and so could persist longer on a surface. Another potential advantage concerns mixtures. Human DNA is the same regardless where it comes from,i.e., skin or saliva, and this can cause problems when analyzing mixtures. Whereas, the bacteria found in saliva is different from bacteria found on skin (Costello et al., 2009). Thus, it is reasonable to think that it could be possible to extract the salivary microbiome profile of one person from the skin microbiome profile of another. However, if a mixture was formed from the same trace type then mixture analysis will clearly increase the complexity of the evaluative task.

A combination of PCR and high throughput sequencing is used to analyze these types of traces. Specifically, a target sequence is chosen which can, after analysis, be used to distinguish as many bacterial taxa as possible. The most commonly used target is 16SrRNA, however, a combination of targets may produce more detail and hence a more accurate picture of the microbiome. After the sequences have been quality filtered and then clustered together the final dataset produced is in the form of a table containing bacterial species abundance for each trace or target (if more than one target is analyzed) and the taxa name. This table can then be used for all downstream analysis/interpretation. One drawback of high throughput sequencing is the number errors. Unlike standard DNA typing which uses one round of PCR followed by capillary electrophoresis, high throughput sequencing uses 2 rounds of PCR, one to amplify specific targets and one during the sequencing process. To try and overcome this when the data is quality filtered a certain number of sequences are removed according to a chosen threshold. The questions then posed are: what threshold should be chosen to remove as many erroneous sequences as possible without impeding downstream analysis and how to incorporate this into data interpretation?

The interpretation of microbiome data for the purpose of forensic science has not yet been addressed. Forensic science is different to most other science in that the final results have to be presentable to a court and therefore, understandable to lay people. This is where inference and statistics come into play. For standard DNA typing, current practice focuses on a likelihood ratio (LR) assignment based mainly on allele proportions for the relevant population. This is used when the court is interested in discriminating between hypotheses relating to the source of the recovered stain. The allele proportions are calculated by analyzing a certain number of people from the relevant population. These population specific data enable an acceptably accurate measure of the rarity of a DNA profile. Behind these allele proportions is a well-understood model of inheritance, which forms the backbone of all calculations. Furthermore, to make this measure as independent as possible all the STRs used are either on different chromosomes or so far apart that linkage is very unlikely. With microbiome data this is more difficult to achieve. Over 700 bacterial species have been found in the mouth (Parahitiyawa et al., 2010) and it is inevitable that some of these species will be co-dependent (Lamont and Jenkinson, 2010). The question then becomes: how is one to account for such data to determine a probabilistic measure to discriminate between the hypotheses of interest? If it is possible to characterize the rarity of a microbiome profile using, for example, the presence/absence of species then a similar method to that used for standard DNA typing could be employed. However, this would involve analyzing a large number of samples to get accurate figures for the proportions of bacteria in the relevant populations. With the current costs of high-throughput sequencing this is not a feasible option. As the cost of analysis decreases more samples can be analyzed for less and this technique may become more viable. There has been an increased interest in microbiome analysis in dentistry (Aas et al., 2005) so in the future it might be possible that everybody will have their oral microbiome analyzed for such a purpose and hence more accurate population proportions for species could be obtained.

A second approach could focus on data from populations and their use for classification to support relatedness to a given cluster. In this context, a question of interest is whether a given trace, say X, fits into either cluster A or cluster B, for example. It then becomes an issue for the scientist to give a value to such an association. The intra- and inter-variability of microbiomes (i.e., the variation for a given person and the variation between different people) play a fundamental role in this task. Previous studies have shown that for both skin and saliva bacterial communities intra-variability is smaller than inter-variability (Fierer et al., 2010; Lazarevic et al., 2010). Therefore, it should be possible to cluster samples, taken from the same person, together, and to support a distinction with respect to samples taken from a different person. However, it also appears relevant to extend research to additional factors, such as diet, antibiotic use, and smoking habit, because these factors can affect microbiome composition.

The challenges associated with this technique are 2-fold: the first relate to the stability of the saliva microbiome and the second to the sequencing method used. The saliva microbiome has been shown to be relatively stable over time (Costello et al., 2009) however, is relative stability good enough for forensic use? More research needs to be carried out to investigate the effect additional factors have on both short term and long-term microbiome stability. One could suppose that the effect of smoking for example would be continuous as long as the person smoked regularly and therefore, would not affect the overall stability. However, for someone with a sporadic smoking habit the effect could be more pronounced. A recent study has shown that people who live together share certain bacteria with each other and their pet dogs (Song et al., 2013). Therefore, knowledge of a person's lifestyle would be very useful when interpreting data. However, these additional factors could also help to discriminate two people with different lifestyles, for example, if a number of canine bacteria were found this would indicate that the person has a pet dog providing additional information to law enforcement agencies when searching for a suspect. As mentioned above there are errors associated with the sequencing method used mainly due to the two rounds of PCR. These errors principally impact upon the rare microbiome i.e., rare bacteria that are represented by only a few sequences. Consequently, how can one differentiate rare bacteria from sequencing errors? For forensic purposes I think the best option is to be conservative and remove most of the rare microbiome helping to ensure as many errors as possible have been removed.

To implement this technique into real casework the additional factors mentioned above need to be investigated and an evaluative framework developed. At the equipment level, with the advances in sequencing technologies and their rising popularity bench-top high-throughput sequencing machines have been developed making this technique more affordable and accessible. The development of a standard operating protocol would enable the exchange of data between laboratories and consequently a database could be built. Once the evaluative framework has been developed this technique could start to be used for cases where all other options have been exhausted, potentially helping with human identification and/or lifestyle indicators.

In conclusion, microbial analysis of body sites could provide additional information where conventional human DNA analysis has failed. However an appropriate evaluative framework needs to be established to interpret the resulting data. Due to the nature of the experiments, and the questions to be asked, it seems reasonable to suggest that current statistical inferential methods could provide the necessary frameworks of thinking to streamline the analysis route.
